# High Expression and Clinical Significance of Elafin in Colorectal Cancer

**DOI:** 10.1155/2019/4946824

**Published:** 2019-06-09

**Authors:** Yun Liu, Yu Tian, Ting Wu, Yun Dai, Weihong Wang, Guigen Teng

**Affiliations:** Departments of Gastroenterology, Peking University First Hospital, Beijing, China

## Abstract

**Objective:**

The decrease of Elafin is associated with several inflammatory diseases. Exogenous Elafin may be a treatment for IBD. Little data has shown the expression of Elafin in patients of colorectal cancer. Here, we tried to explore Elafin expression in human tissues of colorectal cancer.

**Methods:**

We examined the protein expression of Elafin in human tissues of adjacent nontumor and colorectal tumor by immunohistochemistry (IHC) or quantitative real-time polymerase chain reaction (qRT-PCR), then analyzed the clinical and RNA-seq data presented in The Cancer Genome Atlas (TCGA) database to confirm the relationship between Elafin levels and colorectal tumor.

**Results:**

Of the 88 paired samples, 68 colorectal cancer tissues indicated a high expression of Elafin compared with 52 matched adjacent noncancerous tissues. And the mRNA levels of Elafin in 35 paired tissues showed a similar trend. The RNA-seq and clinical data were available in 438 colorectal cancer tissues and 41 normal tissues in TCGA database. The RNA-seq data showed that Elafin mRNA was upregulated about twofold in colorectal cancer samples as compared to adjacent noncancerous samples (176.42 ± 402.13 vs. 96.75 ± 150.07; *P* = 0.208). No statistically significant correlation was found between the Elafin expression and the age, gender, tumor invasive stage, lymph node metastasis, and distant metastasis both at the protein and mRNA levels. However, the Elafin expression was correlated with clinical stage based on the AJCC guidelines at protein levels but not mRNA levels.

**Conclusions:**

Elafin was upregulated in patients of colorectal cancer, resulting to potential limitations for exogenous Elafin treatment.

## 1. Introduction

Inflammatory bowel disease (IBD) is a chronic nonspecific inflammatory disease, including ulcerative colitis (UC) and Crohn's disease (CD). Recently, an increasing morbidity of IBD has shown in Asian populations, which was the low-incidence area previously [1]. The main treatment of IBD was anti-TNF-*α* therapy, but 40% of patients did not respond [2]. Based above, it is crucial to seek a protective and effective therapeutic method.

The key point of pathogenesis mechanism in IBD was still unclear, but combined effect of genetic, immune, environmental, and microbial factors was widely accepted [3]. Previous studies have confirmed that proteases and protease inhibitors play an important role in chronic inflammation [4–5]. Elafin is a low molecular weight antiproteinase, which antagonizes human neutrophil elastase (NE), pancreatic elastase, proteinase 3, and endogenous vascular elastase and also has several functions, such as anti-inflammation, immune regulation, antimicrobe, antiproliferation, vascular remodeling, and wound healing [6]. The decrease of Elafin is associated with several inflammatory diseases [7]. Recently, we also found that Elafin reduced in active IBD patients and was correlated with disease activity negatively [8]. Furthermore, several studies confirmed that probiotic-expressed Elafin protected against inflammation and restored homeostasis in animal models of colitis [9–11], suggesting that exogenous Elafin may be a treatment for IBD. However, differential expressions of Elafin have been reported in several tumors. Elafin was overexpressed and secreted by basal-like breast cancer and ovarian cancer, leading a proliferative effect through the MAP kinase pathway [12]. Elafin was increased and correlated with the differentiation of esophageal cancer [13]. In contrast, Elafin is downregulated in ductal carcinoma in situ, invasive breast tumor, ovarian cystadenoma, borderline ovarian tumor, and invasive ovarian cancer [14]. Therefore, the safety of engineered probiotic-expressed Elafin needed to be estimated, but little data have showed the Elafin expression in colorectal carcinoma, which could illuminate its role in colorectal tumors.

In this study, we tried to explore the Elafin expression in human tissues of colorectal cancer, which was never investigated previously. We examined the protein expression of Elafin in human mucous tissues of adjacent nontumor and colorectal tumor by immunohistochemistry (IHC). Then, we analyzed the clinical and RNA-seq data presented in The Cancer Genome Atlas (TCGA) database to confirm the mRNA levels of Elafin and relationship between Elafin and colorectal tumor.

## 2. Materials and Methods

### 2.1. Clinical Samples

Ninety patients with colon cancers were enrolled, who were diagnosed with histopathology after the resection of colon cancer at Peking University First Hospital from 2017 to 2018. A pair of biopsy samples of the colon cancer tissues and adjacent nontumor tissues was collected from every patient, stored at -80°C after frozen in liquid nitrogen. And the clinical information in these patients was gathered, including age, gender, clinical stage, tumor invasive stage, lymph node metastasis, and distant metastasis. All of the tumor samples (*N* = 90) and adjacent nontumor tissues (*N* = 90) were used for tissue microarray analysis to investigate the expression of Elafin protein, but only eighty-eight pairs of patients were used for statistics, due to missing clinical information. Of all colon samples, 35 paired tissues were used for quantitative real-time polymerase chain reaction (qRT-PCR). The patients included 50 males and 38 females, ranging in age from 32 to 91 years. This study was approved by the ethical committee of First Hospital of Peking University, and every patient has provided written informed consent (No. 20171376).

### 2.2. Immunohistochemical Analysis

Tissue specimens were fixed by 10% neutral formalin and embedded by paraffin. The sections inhibited endogenous peroxidase activity by 3% hydrogen peroxide, after deparaffinization and rehydration. And the tissues were incubated with rabbit anti-human Elafin (1 : 100; Santa Cruz, CA) at 4°C overnight. After incubation with anti-rabbit IgG horseradish peroxidase (1 : 100; Dako, Glostrup, Denmark) at room temperature for 1 hour, the 3,3′-diaminobenzidine (DAB) was used for visualization of the Elafin expression. Following counterstain with hematoxylin, the sections were observed in an optical microscope.

Immunostaining scores of Elafin were accomplished by two experienced investigators independently. The score of staining intensity was as follows: 1 (negative brown), 2 (weak brown), 3 (moderate brown), and 4 (strong brown). The staining extent was based on the rate of positive cells: 0 (0-5%), 1 (6-25%), 2 (26-50%), 3 (51-75%), and 4 (76-100%). The final score was defined as the sum of the intensity and extent of the scores. When discrepancy in the final score was encountered, it was resolved by discussion between two investigators. The specimens were separated into two groups based on the final score: high (>4) and low (≤4).

### 2.3. RNA Extraction and qRT-PCR

Total RNA from frozen colon tissues was isolated using TRIzol reagent (Invitrogen, Carlsbad, CA, USA). The quality and concentration of RNA were determined using a NanoDrop 2000 spectrophotometer (Thermo Fisher Scientific, MA, USA). Then, a Reverse Transcriptase Kit (TaKaRa Biotechnology Group, Dalian, China) was used to generate cDNA. qRT-PCR was conducted on the Applied Biosystems 7500 Real-Time PCR System with SYBR Green Master Mix (Thermo Fisher Scientific, Grand Island, NY, USA). The sequences of primers were as follows: Elafin forward: 5′-CTTCTTGA- TCGTGGTGGTGTTC-3′ and reverse: 5′-AACGGGATCTTGTCCATTGAAT-3′, and glyceraldehyde 3-phosphate dehydrogenase forward: 5′-GCCTGGTCACCA- GGGCT-3′ and reverse: 5′-AATTTGCCATGGGTGGAATC-3′.

### 2.4. Analysis of Colorectal Cancer Data from TCGA

The clinical and RNA-seq data of the 459 colorectal cancer patients and 41 normal tissues were obtained from TCGA database (https://cancergenome.nih.gov/). The clinical data was an abstract from TCGA database, including age, gender, clinical stage, tumor invasive stage, lymph node metastasis, and distant metastasis. The patients with integrated clinical information and mRNA levels of Elafin were enrolled, including 438 colorectal cancer samples and 41 normal tissues finally. The data used in this paper are presented to the public without limitation and restriction. The mRNA expression level of Elafin was analyzed in subjects with different clinicopathological features. The differential expression genes in the cancer samples compared with normal tissues were conducted.

### 2.5. Statistical Methods

#### 2.5.1. Immunostaining Score

The proportion of a high-score group was calculated. And the paired Chi-square test was applied to analyze the proportions between tumor and nontumor tissues in all and in different clinicopathological characteristics. The relationship between the percentage of a high-score group and the clinicopathological variables was calculated by the Chi-square test or Fisher's exact test. The survival analysis was compared between different protein levels of Elafin groups by Kaplan-Meier curves and a log-rank test.

#### 2.5.2. RNA Data from qRT-PCR or TCGA Database

Continuous variables were displayed as mean and standard deviation. The relationship between RNA-seq levels of Elafin and clinicopathological features was conducted by an independent two sample *t*-test. A two-tailed *P* value of less than 0.05 was defined as statistically significant difference. The SPSS 20.0 software was used for statistical analyses. And the analysis of differential expression genes was through EBSeq.

## 3. Result

### 3.1. Expression of Elafin in Colorectal Cancer Tissues and Nontumor Normal Tissues

**Figure 1:**
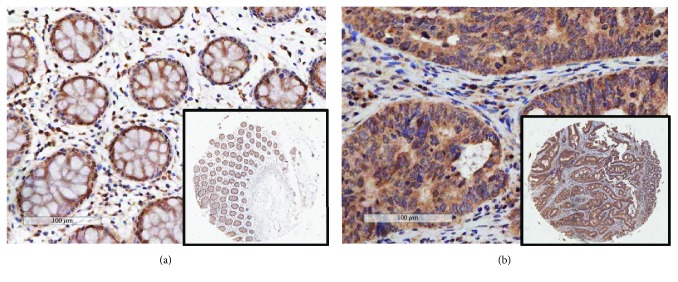
The expression of Elafin in colorectal cancer and nontumor tissues. (a) Elafin expression in normal colonic mucosa epithelium by IHC. (b) Elafin expression in colon cancer tissues by IHC.

Eighty-eight paired human cancer tissues and nontumor tissues were selected for immunohistochemical analysis. The subjects were 50 males and 38 females, with an average age of 66.86 ± 10.82, ranging in age from 32 to 91 years. Of the 88 paired samples, 68 (77.3%) colorectal cancer tissues indicated a high expression of Elafin compared with 52 (59.1%) matched adjacent noncancerous tissues (Table 1, *P* = 0.010; Figures 1(a) and 1(b)). And 35 pairs of tumor and nontumor samples from the same cohort were measured by qRT-PCR. Colorectal cancer tissues showed higher mRNA levels of Elafin than nontumor tissues (Table 1, *P* = 0.039). In addition, the RNA-seq data of 438 colorectal cancer tissues and 41 normal tissues were analyzed. Results showed that Elafin was upregulated about twofold in colorectal cancer samples as compared with nontumor tissues (Table 1, *P* = 0.208). The result suggested that the Elafin expression in colorectal cancer patients was increased at the protein and mRNA levels, compared with nontumor colon tissues by immunohistochemical scoring and analysis of TCGA database.

### 3.2. Expression of Elafin in Colorectal Cancer Patients in Different Clinicopathological Features Compared with Nontumor Tissues

Based on a high expression of Elafin in tumor tissues compared with nontumor tissues, eighty-eight paired human tissues were divided by different clinicopathological parameters, including clinical stage, tumor invasive stage, lymph node metastasis, and distant metastasis. A high expression of Elafin evaluated by immunostaining scoring was observed in all subgroups. However, the expression of Elafin between cancer samples and matched adjacent nontumor tissues in some subgroups showed no significant differences, including stage III-IV of clinical stage (*P* = 0.076), stage T4 of tumor invasive (*P* = 0.18), stage N1-N2 of lymph node metastasis (*P* = 0.078), and stage M1 of distant metastasis (Table 2). Systemic bioinformatics analysis of RNA-seq data of colorectal cancer samples from The Cancer Genome Atlas (TCGA) was performed. Differential expression analysis between 438 colorectal cancer tissues and 41 nontumor tissues showed high Elafin mRNA levels in cancer samples and matched adjacent nontumor samples in all cases, except some subgroups without statistically significant differences, i.e., stage III of clinical stage, stage T4 of tumor invasive, and stage N1 of lymph node metastasis (Table 3).

### 3.3. The Relationship between Elafin Expression and Clinicopathological Features in Colorectal Cancer Patients

The relationship between Elafin expression and clinicopathological features was analyzed by immunostaining scores and mRNA data of colorectal cancer patients. The 77.27% (68/88) cancer tissues revealed high scores of immunohistochemical method. As shown in Table 4, no statistically significant correlation was found, both at protein and mRNA level, between the Elafin expression and the age, gender, tumor invasive stage, lymph node metastasis, and distant metastasis. However, Elafin expression was correlated with clinical stage based on the American Joint Committee on Cancer (AJCC) guidelines at a protein level (*P* = 0.028) but not mRNA level (*P* = 0.537).

### 3.4. Survival Analysis of Elafin Expression in Colorectal Carcinoma Patients

**Figure 2:**
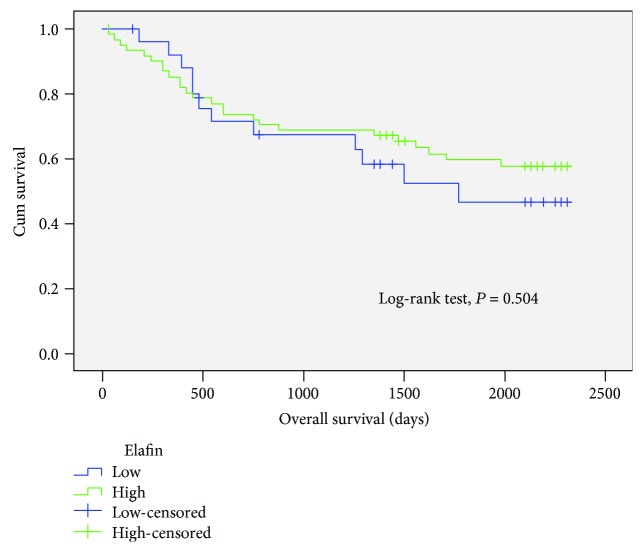
Survival analysis in 88 colorectal cancer patients between high and low expression of Elafin. The mean overall survival time was 1530.95 ± 170.05 days in the low-expression group compared with 1636.74 ± 112.44 days in the high-expression group.

The overall survival (OS) was compared between high and low protein levels of Elafin in 88 colorectal cancer patients. The mean overall survival time was 1530.95 ± 170.05 days in the low-expression group compared with 1636.74 ± 112.44 days in the high-expression group (Figure 2). Nonsignificant differences in the OS between two groups were found (*P* = 0.504).

## 4. Discussion

Elafin, a low molecular weight antiproteinase, was detected in some human epithelial tissues, including bronchial epithelium, intestine epithelium, female genital epithelium, and breast epithelium, and some immunocytes, such as monocytes, alveolar macrophagocytes, neutrophilic granulocytes, and T cells. Elafin have several functions, such as anti-inflammation, immune regulation, antimicrobial, antiproliferation, vascular remodeling, and wound healing [6]. The expression of Elafin is associated with several inflammatory diseases. For example, the increased expression of Elafin was displayed in patients with psoriasis [15]; however, Elafin levels were decreased in subjects suffering from periodontitis [16], bacterial vaginosis [17], asthma [18], and acute respiratory distress syndrome [19]. Recently, we also found that Elafin was decreased in patients of active IBD and correlated with disease activity negatively [8]. The previous results suggested that intestinal protease and antiprotease imbalance might play an important role in IBD patients, which could influence the intestinal inflammation. But another possibility is that the downregulation of Elafin was a consequence in the progression of IBD instead of the cause. Moreover, several studies revealed that exogenous administration of human recombinant Elafin protects against inflammation in animal models. For instance, Vachon et al. [20] found that lung alveolar spaces of mice treated with recombinant human Elafin intranasally reduced LPS-induced neutrophil infiltration and inflammation biomarkers of MIP-2 and KC significantly. Motta et al. [9] engineered lactic acid bacteria-expressed recombinant Elafin and administrated them to mouse models of acute and chronic colitis, which showed the remission of inflammation and the restoration of intestinal homeostasis. Bermúdez-Humarán et al. [21] compared the efficacy of lactobacillus secreting Elafin, IL-10, and TGF-*β*1 in the mouse models of colitis, suggesting that lactic acid bacteria producing IL-10 or TGF-*β*1 only worked on some inflammatory parameters, but administration of Elafin led to a significant reduction of all inflammatory indicators. Based on these results, Elafin delivered by lactic acid bacteria can be an efficient treatment for IBD patients in the future. However, the differential expressions of Elafin have been reported in several tumors, which were even associated with poor outcome, including ovarian cancers [12], breast tumors [14], and esophageal cancers [13]. A question was raised based on these findings; delivery of Elafin by lactic acid bacteria to treat IBD patients is safe or carcinogenic. But little data have showed the expression of Elafin in patients of colorectal cancer, which cannot illuminate its role in colorectal tumors.

Based on what we know, this is the first study to explore the Elafin expression in colorectal cancer. In this study, 77.3% colorectal cancer tissues indicated an increased expression of Elafin compared with 59.1% matched adjacent noncancerous tissues. And the Elafin mRNA was upregulated about twofold in colorectal cancer samples as compared to adjacent noncancerous samples without significance. Several previous studies reported a similar trend in other tumors. Labidi-Galy et al. [12] showed that the Elafin expression of high-grade ovarian carcinoma was upregulated. Yamamoto et al. [13] indicated 73.5% of esophageal cancer patients observed immunoreactivity with Elafin antibody and Elafin expression was associated with differentiation degree. However, Caruso et al. [14] pointed out that the expression of Elafin was decreased in 24% of ductal carcinoma in situ and 83% of invasive breast tumor as compared to normal breast tissues. These results suggested that the pleiotropic expression of Elafin was observed on tumors, which need to be confirmed in different cancers.

Furthermore, our result suggested the Elafin expression was associated with clinical stage based on the AJCC guidelines at protein levels but not mRNA levels. And nonsignificant differences in the overall survival (OS) between high and low groups of Elafin levels were found in this study. This similar trend was also observed in esophageal cancers [13], by the contrast the levels of Elafin drove poor outcome in ovarian carcinoma, glioblastoma, and breast tumor and was related with cell proliferation [12, 14]. Therefore, the Elafin expression changed and the relationship between Elafin levels and clinicopathological factors was also different over various cancers.

Although our data showed that Elafin levels might not affect the progress of colorectal cancer obviously, upregulation of Elafin was observed in cancer tissues, especially in an early stage. Several clinical studies have reflected chronic inflammation status that showed an increasing risk of developing colorectal cancer; the pattern was widely known as inflammation-dysplasia-carcinoma [22]. Elafin expression was increased in an early stage of colorectal cancer, which might be an adaptive elevation with the inflammatory status. But downregulation of Elafin in malignant tumor might be due to the changed inflammatory microenvironment or the exhaustion of Elafin. And we found that Elafin expressed both in epithelium and immune cells in normal colonic mucosa while it was mainly stained in epithelium. In addition to increased secretion of epithelial cells, we need to pay attention to the role of immune cell-related Elafin expression in the progression of colon cancer. But there are some limitations to observe potential sources of Elafin expression. Tumorigenesis impaired the normal structure of colonic mucosa, and pathological sections can only evaluate several sites in colorectal cancer. The role of Elafin expression in the development of colorectal carcinoma needed more fundamental research in the future.

## 5. Conclusion

Elafin was upregulated in patients of colorectal cancer, resulting to potential limitations for exogenous Elafin treatment.

## Figures and Tables

**Table 1:** Expression of Elafin in colorectal cancer patients and nontumor tissues.

Methods	Tumors	Nontumors	Chi-square/*t*	*P* value
IHC				
Total (*N*)	88	88	6.705	0.010
High *n* (%)	68 (77.3%)	52 (59.1%)		
Low *n* (%)	20 (22.7%)	36 (40.9%)		
qRT-PCR				
Total (*N*)	35	35	2.103	0.039
Elafin levels	2.30 ± 0.80	0.61 ± 0.12		
TCGA				
Total (*N*)	438	41	1.260	0.208
Elafin levels	176.42 ± 402.13	96.75 ± 150.07		

Continuous variables are presented as the mean ± SD. IHC: immunohistochemistry; TCGA: The Cancer Genome Atlas.

**Table 2:** Overexpression of Elafin in colorectal cancer patients evaluated by immunostaining scoring in different clinicopathological features.

Group	Tumor					Normal					*P* value
	Total (*N*)	Low *n* (%)		High *n* (%)		Total (*N*)	Low *n* (%)		High *n* (%)		
Total	88	20	22.7	68	77.3	88	52	59.1	36	40.9	0.01
Clinical stage											
I-II	41	5	12.2	36	87.8	41	16	39.0	25	61.0	0.027
III-IV	47	10	21.3	37	78.7	47	20	42.6	27	57.5	0.076
Tumor invasive											
T1-T3	64	11	17.2	53	82.8	64	26	40.66	38	59.4	0.014
T4	24	4	16.7	20	83.3	24	10	41.7	14	58.3	0.18
Lymph node metastasis											
N0	42	6	14.3	36	85.7	42	17	40.5	25	59.5	0.027
N1-N2	46	9	19.6	37	80.4	46	19	41.3	27	58.7	0.078
Distant metastasis											
M0	83	14	16.9	69	83.1	83	34	41.0	49	59.0	0.005
M1	5	1	20.0	4	80.0	5	2	40.0	3	60.0	—

**Table 3:** Overexpression of Elafin mRNA from TCGA in colorectal cancer patients in different clinicopathological features.

Group	Log2FC	LogCPM	*P* value
Total	0.76	8.99	0.03
Clinical stage			
I	0.86	8.86	0.02
II	0.89	9.02	0.01
III	0.60	8.76	0.08
IV	0.76	8.75	0.03
Tumor invasive			
T1	1.44	8.58	0.00
T2	0.70	8.71	0.05
T3	0.75	8.99	0.03
T4	0.71	8.70	0.06
Lymph node metastasis			
N0	0.86	9.03	0.01
N1	0.28	8.43	0.36
N2	1.13	9.09	0.00
Distant metastasis			
M0	0.83	9.03	0.02
M1	0.76	8.75	0.03

**Table 4:** Correlation of Elafin expression with clinicopathological characteristics of colorectal cancer patients.

Clinical feature	IHC					TCGA			
	Total (*N*)	High *n* (%)	Low *n* (%)	*χ* ^2^	*P* value	Total (*N*)	PI3 levels	*t*	*P* value
Age				0.885	0.347			0.086	0.931
<65	36	26 (72.2)	10 (27.8)			167	178.54 ± 374.87		
≥65	52	42 (80.8)	10 (19.2)			271	175.12 ± 418.71		
Gender				0.018	0.892			-0.799	0.425
Male	50	39 (77.6)	11 (22.4)			230	161.82 ± 415.29		
Female	38	29 (76.3)	9 (23.7)			208	192.57 ± 387.41		
Clinical stage				4.849	0.028			0.617	0.537
I-II	41	36 (87.8)	5 (12.2)			248	186.81 ± 424.74		
III-IV	47	32 (68.1)	15 (31.9)			190	162.87 ± 371.21		
Tumor invasive				0.097	0.755			-0.183	0.855
T1-T3	64	50 (78.1)	14 (21.9)			384	175.11 ± 375.95		
T4	24	18 (75.0)	6 (25.0)			54	185.79 ± 558.44		
Lymph node metastasis				3.26	0.071			0.390	0.697
N0	42	36 (85.7)	6 (14.3)			257	182.72 ± 417.92		
N1-N2	46	32 (69.6)	14 (30.4)			281	167.48 ± 379.54		
Distant metastasis				0.901	0.343			0.150	0.881
M0	83	65 (78.3)	18 (21.7)			327	176.37 ± 514.35		
M1	5	3 (60.0)	2 (40.0)			64	184.85 ± 392.55		

Continuous variables are presented as the mean ± SD. IHC: immunohistochemistry; TCGA: The Cancer Genome Atlas.

## Data Availability

The clinical data used to support the findings of this study are included within the article.
